# Effect of TiO_2_ doping on the structure and properties of lithium silicate-based glass-ceramics for potential dental applications

**DOI:** 10.1038/s41598-025-34915-2

**Published:** 2026-01-26

**Authors:** M. A. Marzouk, R. L. Elwan, A. M. Fayad, F. H. Elbatal, M. A. Azooz, M. A. Ouis, A. Kh. Helmy, Y. M. Hamdy

**Affiliations:** 1 Glass Research Department, National Research Centre, 33 EL Bohouth St. (former EL Tahrir st.), Dokki, P.O.12622, Giza, Egypt; 2 Spectroscopy Department, National Research Centre, 33 El Bohouth Street (Former EL Tahrir), Dokki, P.O. 12622, Giza, Egypt

**Keywords:** Lithium silicate, Glass-ceramics, TiO_2_, Microhardness, Dental, Chemistry, Materials science

## Abstract

A series of glass samples with the nominal composition 65SiO_2_ - (22.5-x) Li_2_O − 12.5Al_2_O_3_ - xTiO_2_, where x varies as 2.5, 5, 7.5, and 10 mol%, were synthesized using the conventional melt-quenching technique. Differential scanning calorimetry (DSC) was utilized to identify crucial thermal transitions, which informed the process of fabricating corresponding glass-ceramic derivatives. X-ray diffraction (XRD) analysis confirmed the formation of three primary crystalline phases in the glass-ceramics: lithium disilicate (Li₂Si_2_O_5_), lithium aluminosilicate (LiAlSiO_4_), and brookite (TiO_2_). Scanning electron microscopy (SEM) combined with energy-dispersive X-ray spectroscopy (EDAX) demonstrated that crystal growth increased in size and developed well-defined morphologies. Vickers microhardness testing indicated that TiO_2_-doped lithium silicate glasses and their glass-ceramic counterparts exhibit mechanical properties compatible with dental application requirements. Differential scanning calorimetry (DSC) analysis revealed that increasing TiO_2_ content (2.5–10 mol%) shifted thermal transitions to higher temperatures, indicating improved thermal stability and a stronger glass network. Higher TiO_2_ also enhanced microhardness (5.02–5.93 GPa) and compressive strength (440–542 MPa), with further gains after heat treatment due to TiO_2_-induced crystallization of hard phases. Corresponding glass-ceramics showed increased hardness (5.51–7.27 GPa), compressive strength (492–583 MPa), and density (2.478–3.441 g/cm³), confirming the reinforcing and densifying effects of TiO_2_. Fourier transform infrared spectroscopy (FTIR) results suggested that modifiers such as Li_2_O and TiO_2_ disrupt the SiO_4_ tetrahedral network by introducing non-bridging oxygens (NBOs) and weakening some bonds, thereby affecting network polymerization and structural rigidity. TiO₂ incorporation enhanced thermal stability, hardness, and compressive strength, with further gains after heat treatment due to TiO_2_-induced crystallization. FTIR analysis confirmed structural modifications promoting a stronger glass network. These improvements yield glass-ceramics with mechanical and thermal properties comparable to dental enamel, enhancing their suitability for restorative applications.

## Introduction

Lithium silicate glass and glass-ceramics have emerged as highly promising candidates for biomaterials applications, particularly in the field of dentistry, due to their unique combination of mechanical, aesthetic, and biological properties. Over the past decades, the evolution of lithium silicate-based glass-ceramics has revolutionized restorative and prosthetic dental treatments, offering outstanding esthetic qualities that closely mimic natural tooth structure while maintaining excellent chemical durability and inertness in the oral environment^1–3^. These materials are engineered to possess a microstructure that enhances both strength and translucency, making them ideal for dental crowns, bridges, veneers, and other restorative applications where both function and appearance are critical^4–6^.

The biocompatibility of lithium silicate glass-ceramics is a significant factor in their suitability for dental use. Studies have demonstrated that these materials exhibit minimal cytotoxicity, promote cellular adhesion and proliferation, and can facilitate the formation of a mineralized matrix, which is essential for integration with natural tissues^2,7^. Furthermore, incorporating lithium ions into bioactive glass matrices enhances their bioactivity. This promotes the formation of hydroxyapatite layers on the glass surface. Hydroxyapatite is essential for bonding with natural bone. As a result, these materials support hard tissue regeneration more effectively. This innovation improves the potential for bone repair applications^7^. The mechanical properties of lithium disilicate glass-ceramics, including high flexural strength and fracture toughness, are attributed to their controlled crystallization processes and the presence of rod-like or whisker-reinforced microstructures, which deflect cracks and improve durability under masticatory forces^7^. In addition to their mechanical and biological advantages, lithium silicate glass-ceramics offer superior processability, allowing for precision manufacturing through CAD/CAM technologies and additive manufacturing, which enables the creation of custom-fit dental restorations with consistent quality and performance. The elastic modulus of lithia silicate-based ceramics is well-matched to that of natural tooth structures, reducing the risk of failure and enhancing the longevity of dental restorations. Their chemical stability ensures resistance to degradation in the challenging oral environment, while their translucency and color stability contribute to highly aesthetic outcomes^1,4^. The ongoing development and clinical adoption of lithium silicate glass and glass-ceramics underscore their transformative impact on dental biomaterials science, positioning them at the forefront of modern restorative dentistry. Their ability to combine strength, aesthetics, biocompatibility, and processability makes them exceptional materials for current and future dental applications, with continued research likely to expand their use in regenerative and personalized dental therapies^3,8–10^. The incorporation of titanium dioxide (TiO_2_) into silicate, phosphate, and borate glasses has garnered significant attention for bio-glass ceramics. TiO_2_ acts as both a network modifier and a network former within these glass matrices, profoundly influencing their physicochemical, mechanical, and biological properties. In silicate-phosphate glasses, the addition of TiO_2_ enhances thermal and mechanical stability, increases the processing window, and promotes the formation of interconnected microporosity, which is critical for tissue integration and regeneration^11–13^.

The addition of TiO_2_ raises the activation energy for crystallization, enhancing glass resistance to devitrification. This makes it easier to fabricate porous scaffolds ideal for dental implants and bone regeneration. TiO_2_ - doped silicate-phosphate glasses also show improved cell viability. They enable controlled release of Ti^4+^ ions, which stimulate beneficial cellular responses. These properties make TiO_2_ glasses promising for dental tissue engineering^12,13^. For borate glasses, TiO_2_ incorporation leads to notable improvements in chemical durability, bioactivity, and antimicrobial properties. At lower concentrations, TiO_2_ acts as a network former (TiO_4_ units), while at higher concentrations, it serves as a network modifier (TiO_6_ units), resulting in enhanced structural integrity and long-term stability. These modifications not only improve the glass’s suitability for drug delivery and tissue engineering but also contribute to its semiconducting and dielectric properties, which may be advantageous for multifunctional dental materials^12–14^. Additionally, TiO_2_-doped borate glasses exhibit a coefficient of thermal expansion closer to that of metallic dental implants, reducing interfacial stresses and improving the longevity of coatings and restorative materials^15–17^. In the field of dental applications,

TiO_2_-containing glasses are valued for their superior biocompatibility, antibacterial activity, and ability to promote tissue regeneration. TiO_2_ nanostructures, in particular, enhance the mechanical strength of dental materials, provide photocatalytic and antimicrobial effects, and serve as effective carriers for localized drug delivery. These properties make TiO_2_-modified glasses promising candidates for dental implants, restorative materials, and scaffolds for oral tissue engineering^16–20^.

Despite extensive studies^21–23^ on lithium silicate and lithium aluminosilicate glass-ceramics, the controlled influence of TiO₂ as a functional dopant on their structural evolution, crystallization behavior, and mechanical reinforcement remains insufficiently explored. This work addresses that gap by systematically varying TiO_2_ content (2.5–10 mol%) in a 65SiO_2_-(22.5-x) Li_2_O-12.5Al_2_O_3_ -xTiO₂ system to elucidate its role as a nucleating and network-modifying oxide. Unlike previously reported compositions, this tailored approach demonstrates how TiO_2_ incorporation governs phase formation (Li_2_Si_2_O_5_, LiAlSiO_4_, and brookite), promotes controlled crystallization with refined microstructures, and yields superior hardness and thermal stability properties closely aligned with dental enamel, marking a novel pathway for designing high-performance dental glass-ceramics.

The primary aim of this work is to develop and characterize a novel series of titanium dioxide (TiO_2_)-doped lithium silicate glasses and their corresponding glass-ceramics derived from the base composition 65SiO_2_ - (22.5-x) Li_2_O − 12.5Al_2_O_3_ - xTiO_2_, with systematic variations in the TiO_2_ content. This research introduces a new compositional approach to glass and glass-ceramic design by incorporating TiO_2_ as a functional dopant, exploring its influence on the structural, morphological, and mechanical properties of the resulting materials. The structural evolution and network connectivity of the synthesized glasses were thoroughly examined using Fourier Transform Infrared Spectroscopy (FTIR) to gain insights into bond configurations and vibrational modes, while the phase composition and crystallinity of glass-ceramic forms were investigated using X-ray Diffraction (XRD). Scanning Electron Microscopy (SEM) combined with Energy Dispersive X-ray Analysis (EDAX) was employed to assess the surface morphology, microstructure, and elemental distribution, ensuring homogeneity and identifying the nature of crystallites formed. Beyond structural analysis, the study extends to evaluating mechanical performance through microhardness testing, a key indicator of material stability and resistance to wear, which is particularly relevant in biomedical contexts. The integration of mechanical testing with compositional and morphological studies allows for a comprehensive assessment of the suitability of glasses and their corresponding glass-ceramics for dental applications, where high strength, biocompatibility, and resistance to deformation are crucial.

## Experimental details

### Glass preparations

A series of glass samples with the nominal chemical composition 65SiO_2_ - (22.5 - x) Li_2_O − 12.5Al_2_O_3_ - xTiO_2_, where x takes the values 2.5, 5, 7.5, and 10 mol%, were systematically synthesized using a conventional melt-quenching technique. High-purity reagents (99.9%, procured from Sigma Aldrich) were employed for the preparation of the glass batches. Specifically, lithium carbonate (Li_2_CO_3_) served as the precursor for Li_2_O, while high-purity silica (SiO_2_), Aluminum oxide (Al_2_O_3_) and titanium dioxide (TiO_2_) were utilized directly as received. Accurately weighed quantities of the starting materials were thoroughly mixed and subsequently transferred into platinum crucibles. The mixtures were then subjected to melting at 1400 °C in an electric furnace (VECSTAR UK). The melting process was maintained for two hours, during which the crucibles were periodically rotated to ensure uniform mixing and to promote compositional homogeneity in the melt. Upon completion of the melting stage, the homogeneous molten glass was rapidly cast into preheated stainless-steel molds to form glass samples. Immediately following casting, the glass specimens were transferred to a muffle furnace pre-set at 400 ± 5 °C for annealing. The annealing process was carried out for one hour to relieve internal stresses developed during quenching. After the annealing period, the furnace was switched off and allowed to cool naturally to ambient temperature, with the glass samples remaining inside to ensure gradual cooling and to prevent the formation of thermal stresses.

**Fig. 1:**
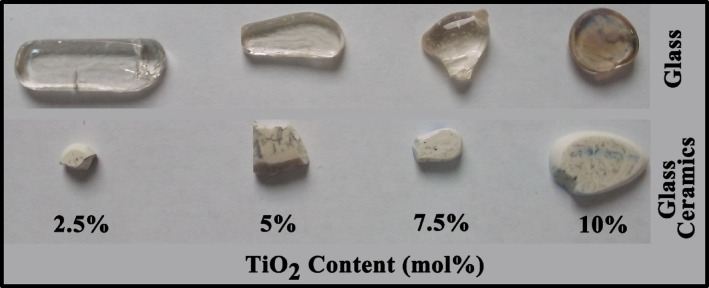
Photographic view of the prepared glass and glass – ceramics.

Figure 1 represents a photographic view of the prepared glasses and their corresponding glass ceramics.

### Characterizations

Differential scanning calorimetry (DSC) was used to identify and analyze the principal thermal events of the synthesized amorphous samples, specifically focusing on the crystallization temperature (T_c_) peak. These measurements were carried out using a Setaram THEMYS ONE + DSC system. During the experiment, powdered glass samples were placed in platinum crucibles, with a separate platinum crucible containing Al_2_O_3_ serving as the reference. All analyses were conducted in an inert atmosphere, utilizing a heating rate of 10 °C per minute under a flow of nitrogen gas.

The crystalline structure of the synthesized glass-ceramic was analyzed and characterized on finely ground powder samples through X-ray diffraction (XRD) patterns obtained using a Bruker diffractometer (Germany) equipped with graphite-monochromatized Cu-Kα radiation source, with a wavelength of 1.5405 Å. The diffractometer was operated at 40 kV and 30 mA, ensuring optimal performance for the analysis. Diffraction data were recorded in the 2θ range.

The microstructure of the developed phases was examined using a scanning electron microscope. A high-resolution SEM (HSEM), specifically the Quanta Field Emission Gun (FEG) 250 model from FEI, equipped with an EDAX system for elemental analysis, was employed. The analysis was conducted at an accelerating voltage of 30 kV, with magnification ranging from 10x to 400,000x, and a spatial resolution of 3.5 nm. To investigate the grain morphology, freshly fractured surfaces of the glass-ceramics were chemically etched in a 5% hydrofluoric acid (HF) solution for 30 s prior to imaging.

Microhardness was evaluated using the Vickers indentation method on finely polished specimens with a Shimadzu microhardness tester (Japan). To ensure accuracy and reliability, a consistent load of 100 g was applied for 15 s at each indentation site. Measurements were taken at five different locations on each sample to provide a representative assessment of material hardness. The average of these readings offered a comprehensive understanding of the microhardness characteristics, yielding valuable insights into the mechanical behavior and surface deformation resistance of the materials.

Fourier transform infrared (FTIR) absorption spectroscopy, performed using a Bruker Vertex 8 V spectrometer (Germany), was utilized to investigate the structural characteristics of both the glasses and their corresponding glass-ceramic derivatives. The measurements were carried out at room temperature over the wavenumber range of 400 to 4000 cm^− 1^. Finely ground powders of the glass or glass-ceramic samples were thoroughly mixed with potassium bromide (KBr) in a 1:100 ratio. This mixture was then pressed under a pressure of 5 tons per square centimeter to form uniform pellets, which were immediately analyzed using the FTIR instrument to record their absorption spectra.

## Results and discussions

### Differential scanning calorimetry (DSC) characteristics

**Fig. 2:**
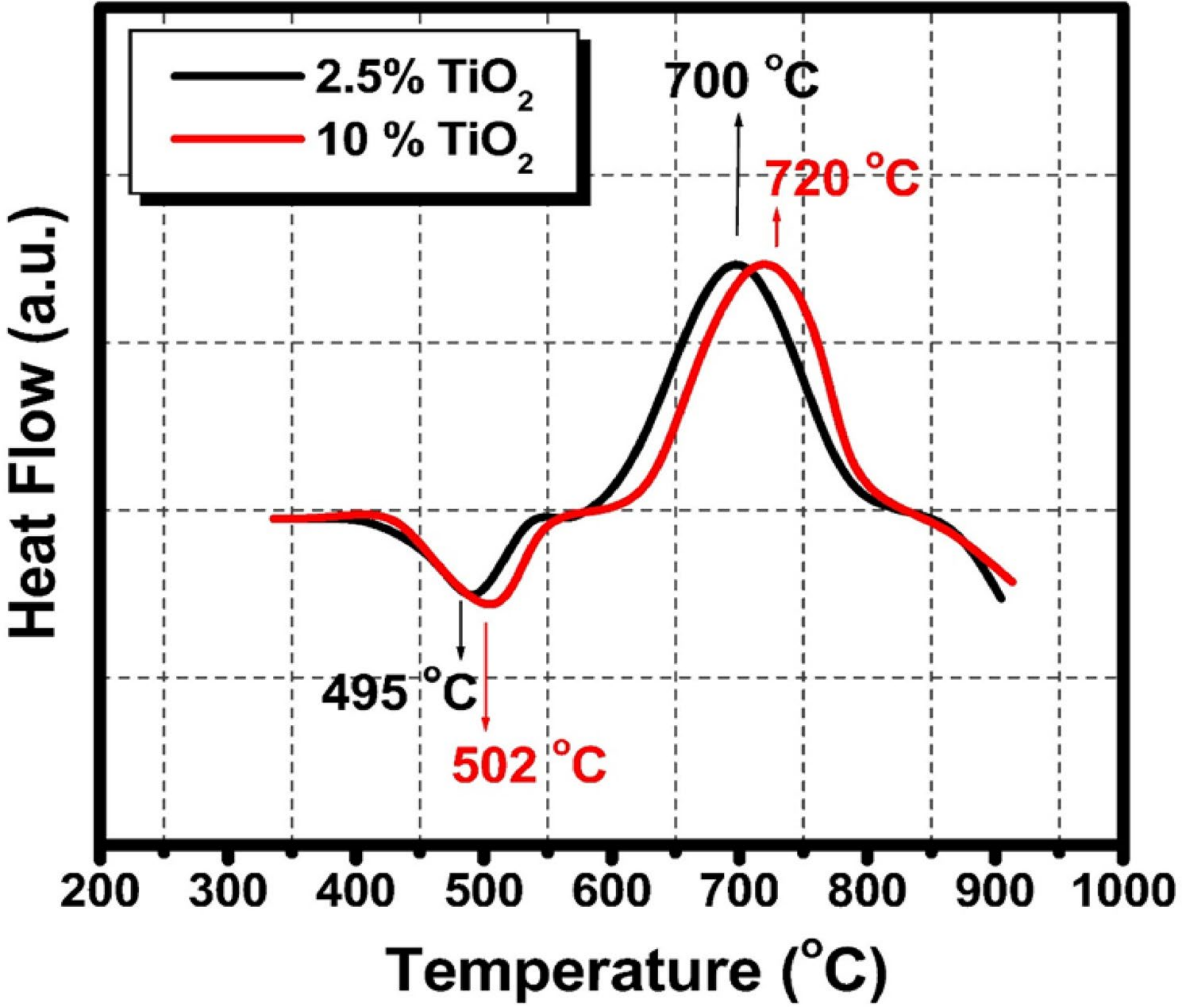
DSC of the prepared TiO_2_ doped glasses where (a) 2.5 mol% and (b) 10% mol % TiO_2_.

Figure 2 represents the DSC analysis of the selected 2.5 and 10 mol% TiO_2_-doped glasses that revealed distinct thermal transitions that vary systematically with TiO_2_ content, showing its significant influence on the thermal behavior of the glass system. For the glass doped with 2.5 mol% TiO_2_, the DSC curve exhibited an endothermic peak (T_endo_) at 495 °C. Following this, an exothermic peak was observed at 700 °C, signifying the onset of crystallization where the amorphous glass matrix begins to reorganize into crystalline phases, releasing latent heat during nucleation and growth. The clear separation between these two peaks suggests a stable thermal window for processing the glass without premature crystallization. In contrast, the glass doped with a higher TiO_2_ content of 10 mol% showed a shift in these thermal events, with the endothermic peak moving to a slightly higher temperature of 502 °C and the exothermic crystallization peak shifting to 720 °C. This upward shift in both T_endo_ and crystallization temperature (T_c_) with increasing TiO_2_ content indicates enhanced thermal stability and a higher resistance to crystallization, which can be attributed to the role of TiO_2_ as a network modifier or intermediate oxide that strengthens the glass structure and raises the energy barrier for atomic rearrangement and nucleation^24–26^. The increase in T_endo_ reflects a more rigid glass network, likely due to the incorporation of TiO_2_ units that restrict the mobility of the silicate network, while the increase in T_c_ suggests that the crystallization process requires higher thermal energy to initiate^25,26^. This behavior is consistent with literature reports that TiO_2_ additions increase both glass transition and crystallization temperatures, thereby improving the thermal stability of the glass and modifying its crystallization kinetics^21–27^. The broader temperature gap between T_g_ and T_c_ in the 10% TiO_2_ glass compared to the 2.5% TiO_2_ sample implies an extended processing window, advantageous for controlled heat treatments designed to customize microstructure and properties. Moreover, the shift in crystallization temperature may also influence the nature and morphology of the crystalline phases formed, potentially affecting the mechanical and functional properties of the resulting glass-ceramics^24–27^.

### XRD pattern characteristics

**Fig. 3:**
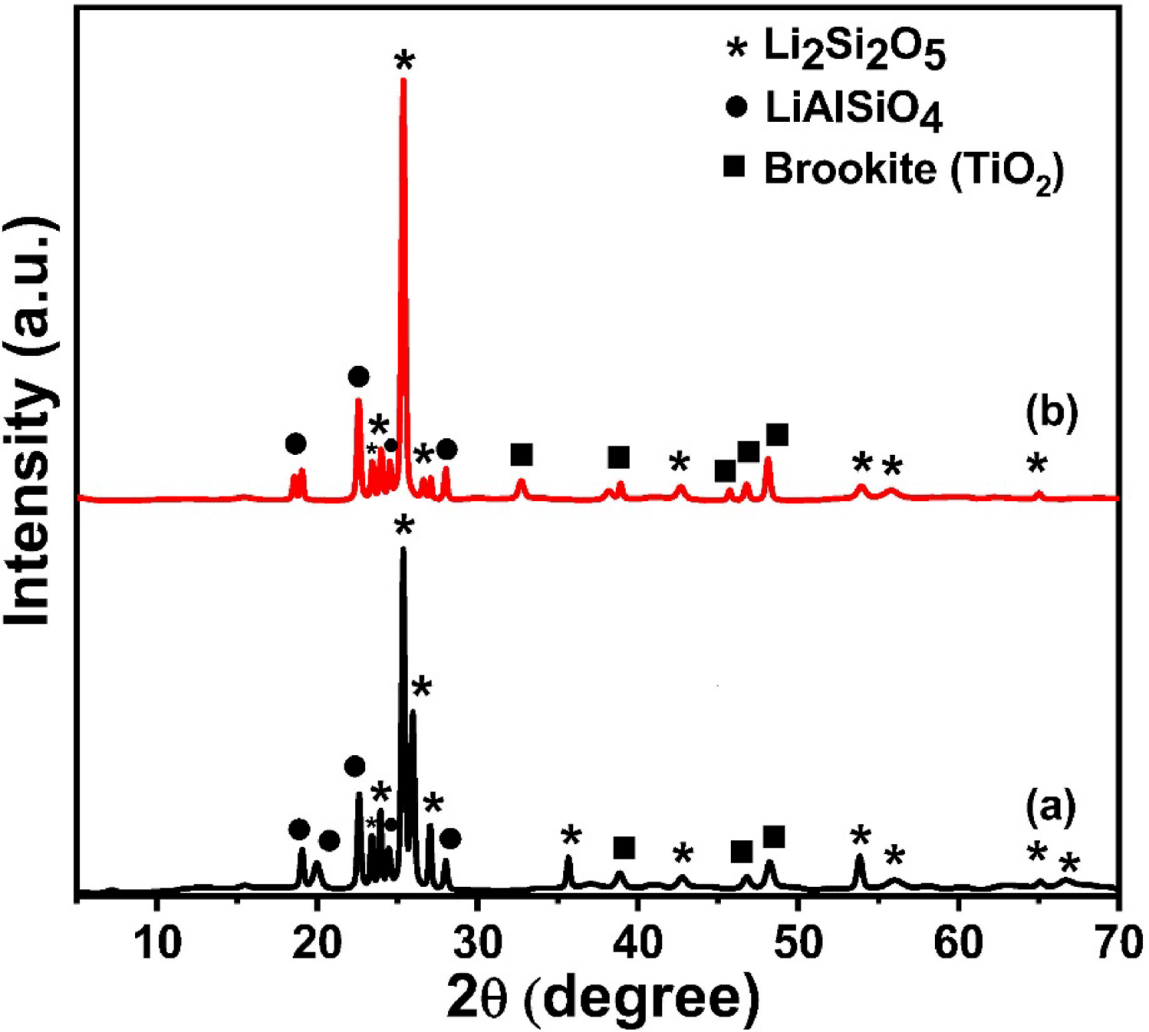
XRD patterns of the prepared TiO_2_-doped glass – ceramics, where (**a**) 2.5 mol% and (**b**) 10% mol% TiO_2_.

Figure 3 depicts the X-ray diffraction analysis that was performed on glass – ceramics that obtained from the parent glass samples with the chemical composition 65SiO_2_ – (22.5-x) Li_2_O – 12.5Al_2_O_3_ – xTiO_2_, where x = 2.5 and 10 mol%. The samples were subjected to a controlled heat treatment at 650 °C for 10 h, with a heating rate of 5 ^o^ C/min. Performing heat treatment slightly below the maximum exothermic peak allows a more controlled nucleation and growth process without rapid or excessive crystallization that might occur at the peak temperature. This can help achieve a more desirable microstructure or phase distribution by slowing down the reaction rate. The XRD patterns obtained reveal the crystalline phases that precipitated from the glass matrix under these conditions. Across all compositions, the XRD patterns indicate the formation of three primary crystalline phases: lithium disilicate (Li_2_Si_2_O_5_), lithium aluminosilicate (LiAlSiO_4_), and brookite (TiO_2_). The identification of these phases was confirmed by matching the diffraction peaks with standard reference cards: Li_2_Si_2_O_5_ (ICDD card no. 96-900-7748), LiAlSiO_4_ (ICDD card no. 96-153-3306), and brookite (ICDD card no.

96-900-4140).

The presence of Li_2_Si_2_O_5_ is evident from its characteristic diffraction peaks, which are prominent in all samples, regardless of TiO_2_ content. This phase is known for its high crystallinity and mechanical strength, which are desirable properties in glass-ceramics^28^. The intensity and sharpness of the Li_2_Si_2_O_5_ peaks suggest a significant degree of crystallization, indicating that the chosen heat treatment parameters are effective in promoting the growth of this phase. LiAlSiO_4_ is also detected in all samples, as confirmed by the matching peaks with the reference card. The formation of this phase is expected due to the presence of both lithium and aluminum in the glass composition. The relative intensity of the LiAlSiO_4_ peaks appears to vary slightly with increasing TiO_2_ content, which may be attributed to the competitive crystallization behavior between the different phases as the glass composition is modified. Brookite, a polymorph of TiO_2_, is identified by its distinctive peaks in the XRD patterns, particularly in samples with higher TiO_2_ content (x = 10 mol%). The emergence and intensification of brookite peaks with increasing x indicate that TiO_2_ acts as a nucleating agent, facilitating its crystallization as well as potentially influencing the crystallization of the other phases. In samples with lower TiO_2_ content (x = 2.5), brookite peaks are present but less intense, suggesting that a threshold concentration of TiO_2_ is required for substantial brookite formation under the applied heat treatment conditions. A systematic comparison of the XRD patterns for different values of x reveals a clear trend: as the TiO_2_ content increases, the intensity of the brookite peaks increases, while the relative intensities of Li_2_Si_2_O_5_ and LiAlSiO_4_ peaks show subtle changes. This suggests that TiO_2_ not only promotes its crystallization but may also influence the nucleation and growth of the lithium silicate and aluminosilicate phases. At higher TiO_2_ concentrations, the competitive crystallization may lead to a slight reduction in the amount or crystallinity of Li_2_Si_2_O_5_ and LiAlSiO_4_, as more TiO_2_ is available to form brookite. The role of TiO_2_ as a nucleating agent is well-documented in the literature^22,29^, and its effectiveness is confirmed by the results of this study. The formation of brookite, rather than other TiO_2_ polymorphs such as anatase or rutile, is particularly interesting and may be attributed to the specific glass composition and heat treatment regime employed. The stabilization of brookite in this system could be of particular interest for tailoring the properties of the resulting glass-ceramics, as brookite has distinct photocatalytic and electronic properties compared to the other TiO₂ polymorphs. The overall crystallization behavior observed in these glass samples is consistent with the known effects of TiO_2_ in silicate glass systems. TiO_2_ serves as an effective nucleating agent, promoting the formation of fine, well-dispersed crystalline phases. The coexistence of Li_2_Si_2_O_5_, LiAlSiO_4_, and brookite suggests a complex interplay between the various components of the glass, with the final phase assemblage being determined by both the initial composition and the thermal history. The formation of multiple crystalline phases can have significant implications for the physical and mechanical properties of the glass-ceramics. For example, the presence of Li_2_Si_2_O_5_ is associated with high strength and toughness, while brookite may impart additional functionalities to the hardness.

### SEM morphology and EDAX analysis

**Fig. 4:**
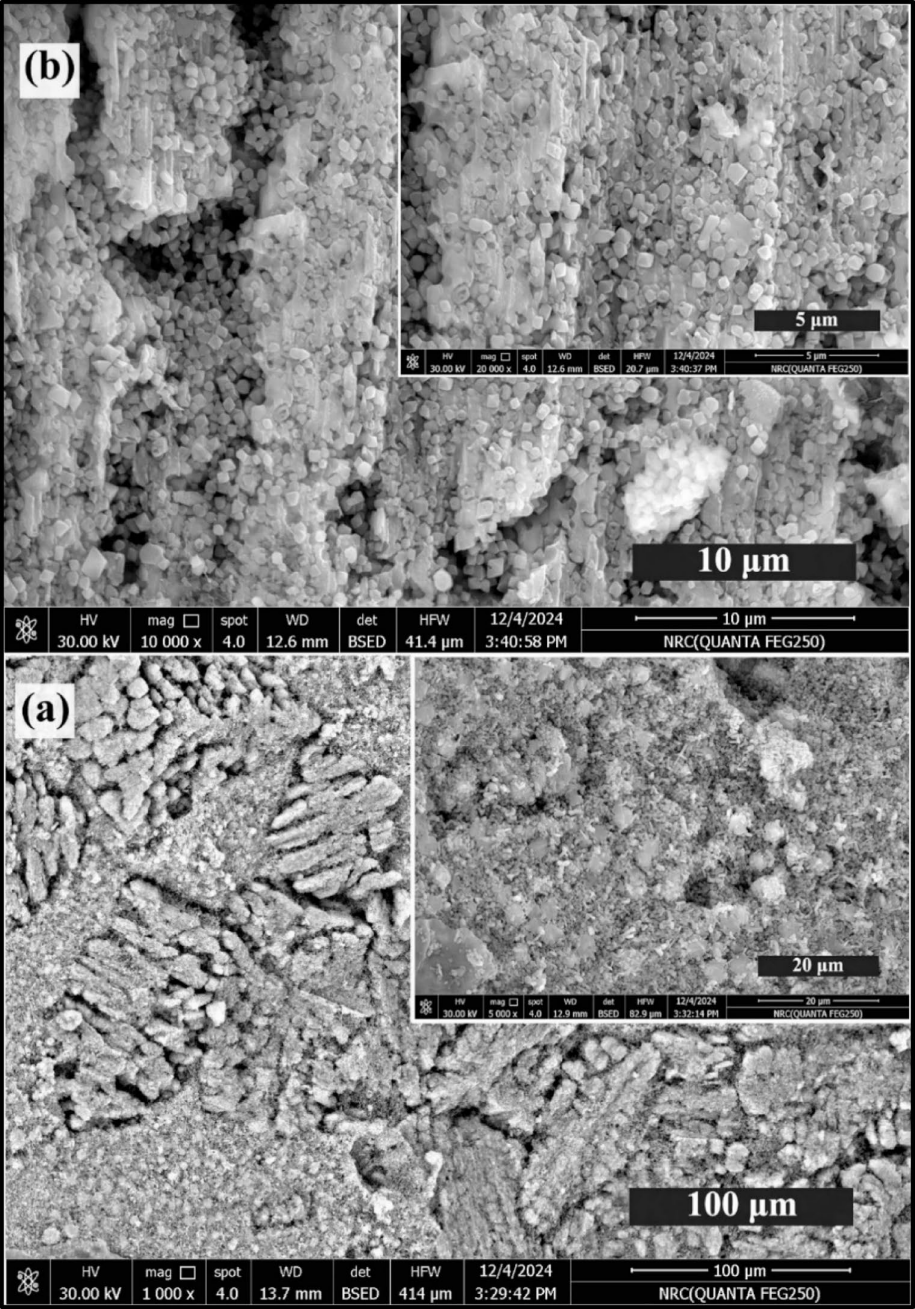
HRSEM of 2.5 mol% TiO_2_-doped glass–ceramics at different scales, where (**a**) 100 and 20 μm, and (**b**) 10 and 5 μm, respectively.

**Fig. 5:**
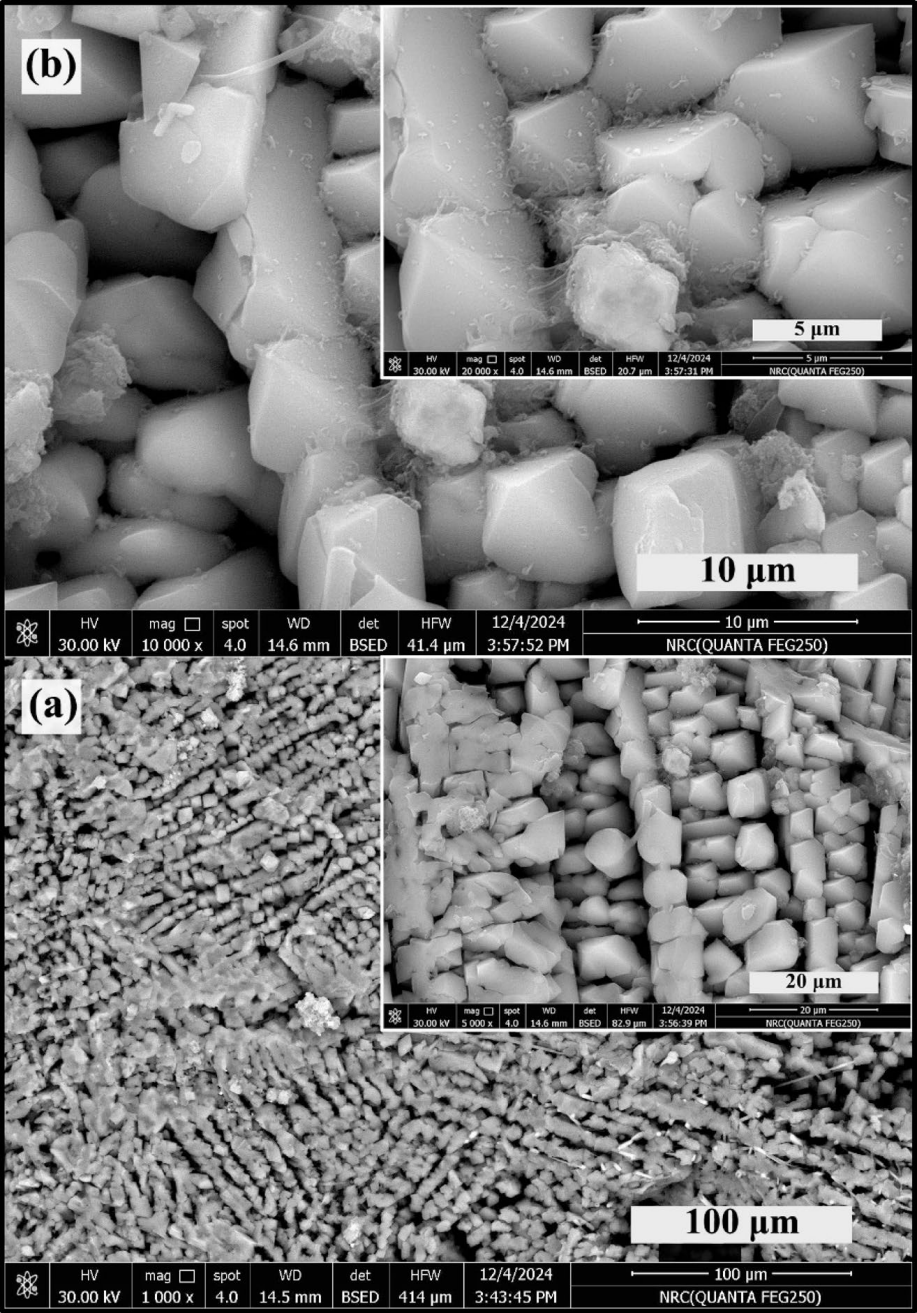
HRSEM of 10 mol% TiO_2_ – doped glass-ceramics at different scales, where (**a**) 100 and 20 μm, and (**b**) 10 and 5 μm, respectively.

Figures 4 and 5 illustrate the micrographs of glass–ceramic samples 2.5 and 10 mol%, revealing a highly regular microstructure predominantly comprised of interlocking sets of lath-like crystals. This intricate arrangement forms a dense and cohesive network, where the elongated laths intersect and intertwine, contributing to the overall structural integrity of the glass-ceramic and its mechanical strength. Upon closer examination at higher magnifications, the lath-like layers exhibit a remarkable transformation in their apparent morphology, with individual segments adopting a square pyramid-like shape. This geometric evolution at the microscale suggests a well-organized crystallization process, where the growth of the crystals is both directional and controlled, leading to the development of these distinctive, faceted features^4^.

The addition of titanium dioxide (TiO_2_) plays a significant role in influencing the microstructural characteristics of the glass–ceramic. As depicted in Fig. 4, increasing the TiO_2_ content markedly affects both the size and morphology of the crystals. Higher concentrations of TiO_2_ promote the formation of larger and more well-defined crystalline phases, altering the aspect ratio and sharpness of the lath-like structures^4,30–32^. This results in a noticeable shift in the microstructural landscape, with the crystals becoming more prominent and their boundaries more distinct. The modification in crystal morphology, driven by TiO_2_, can be attributed to its function as a nucleating agent, which enhances the rate of crystal growth and facilitates the emergence of new structural motifs within the glass–ceramic matrix^30–32^.

**Fig. 6:**
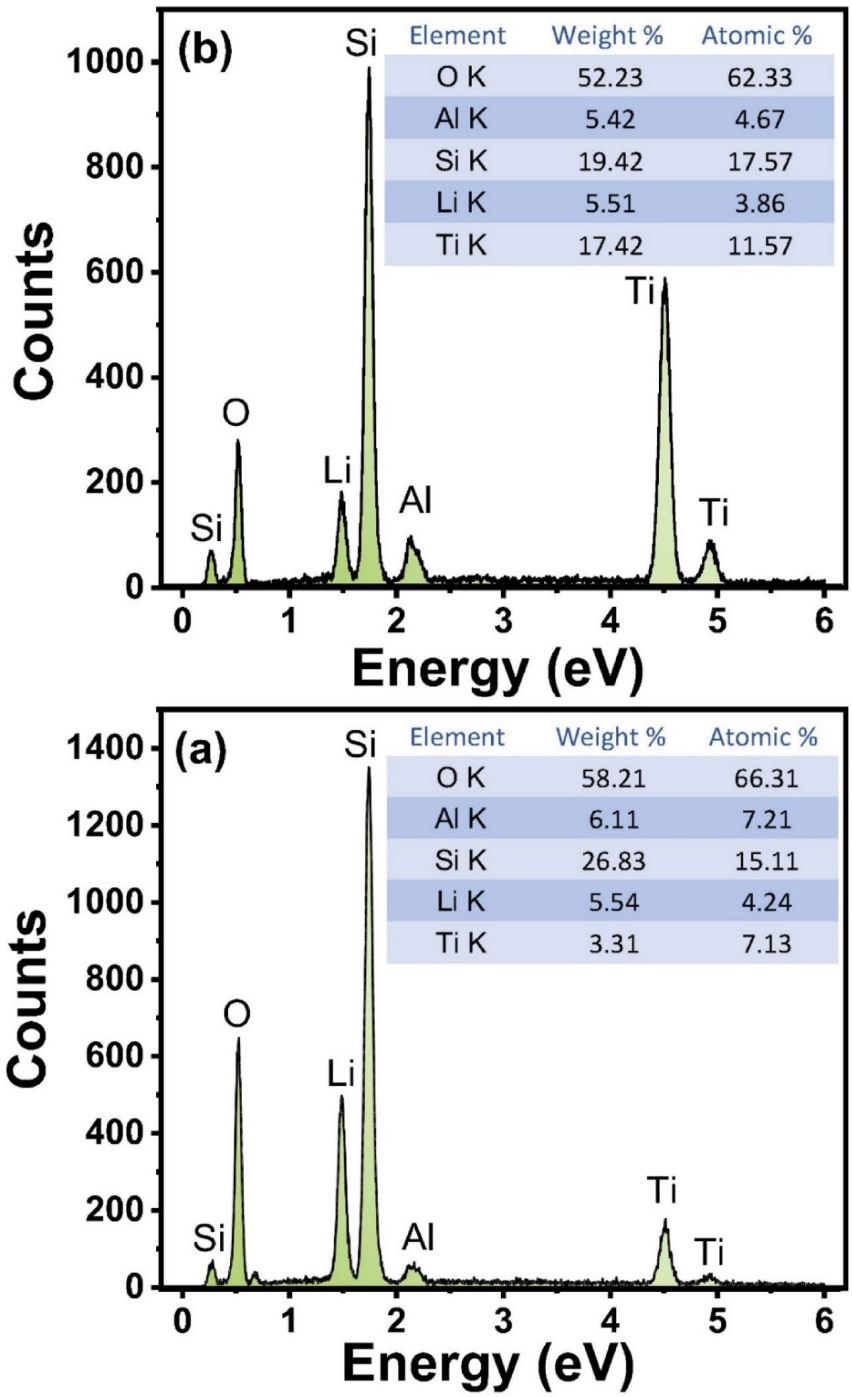
EDAX of the prepared TiO_2_ - doped glass – ceramics where (**a**) 2.5 mol% and (**b**) 10% mol % TiO_2_.

The EDAX spectra depicted in Fig. 6a,b provide a comprehensive elemental profile with their weight and atomic % of the glass–ceramic samples, showcasing prominent spectral peaks for oxygen, silicon, aluminum, and titanium. These elemental signatures are indicative of the principal constituents present within the crystalline phases of the prepared glass-ceramics.

The presence of oxygen and silicon is expected, as they form the backbone of the silicate network that constitutes the glass–ceramic matrix. Aluminum, often incorporated as a network modifier or intermediate, further contributes to the structural complexity and stability of the crystalline phases. The detection of titanium, in particular, is noteworthy; its spectral peak not only confirms its successful incorporation into the glass–ceramic but also aligns with its role as a nucleating agent, which influences the morphology and growth of crystalline domains. The elemental composition revealed by EDAX is in strong agreement with the phase assemblages previously identified through X-ray diffraction (XRD) analysis. XRD patterns have established the existence of specific crystalline phases within the samples, and the corresponding elemental peaks observed in EDAX serve as direct evidence supporting these findings. This correlation between EDAX and XRD results reinforces the reliability of the compositional and structural characterization, providing a robust validation of the material’s phase structure. The convergence of these analytical techniques not only confirms the elemental makeup but also substantiates the interpretation of the phase evolution and crystallization behavior within the glass–ceramic system.

### Microhardness characteristics

**Fig. 7:**
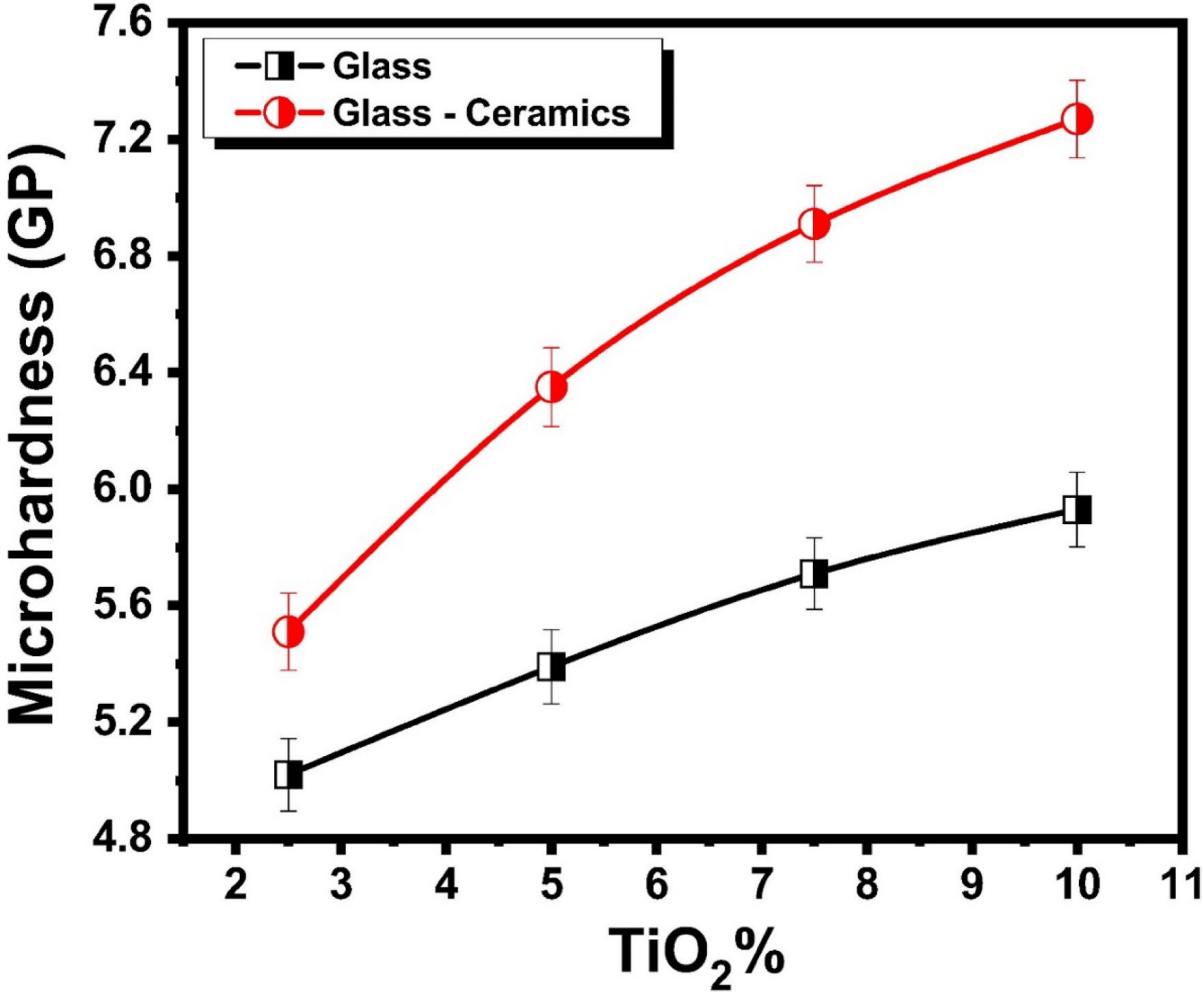
Microhardness of the prepared TiO_2_-doped glasses and their corresponding glass–ceramics.

Figure 7 reveals the microhardness measurements of the prepared glasses and their corresponding glass - ceramic samples. The microhardness measurements of glasses represent a clear trend of increasing with increasing TiO_2_ content. Specifically, the values of the glasses were measured as 5.02, 5.39, 5.71, and 5.93 GPa for the respective TiO_2_ contents 2.5, 5, 7.5, and 10 mol %, indicating that TiO_2_ acts to reinforce the glass network. This increase can be attributed to the role of TiO_2_ that acts as a network former or intermediate oxide, which enhances the connectivity and rigidity of the silicate network by reducing the number of non-bridging oxygens and increasing cross-link density^33–35^. As TiO_2_ replaces Li_2_O, the glass structure becomes more compact and resistant to deformation, leading to higher hardness values. When these glasses are heat-treated to form their corresponding glass-ceramics, the microhardness values increase even further, reaching the range from 5.51 to 7.27 GPa, with increasing the TiO_2_ content from 2.5 to 10 mol % respectively. This pronounced enhancement in the glass-ceramics is primarily due to the nucleating effect of TiO_2_^33–35^, which promotes the controlled crystallization of hard phases such as lithium disilicate and β-spodumene solid solutions^36–40^. The incorporation of TiO_2_ into the glass matrix at concentrations of 2.5, 5, 7.5, and 10 mol% has a significant influence on the crystallization behavior and structural development of the resulting glass-ceramics. As the TiO_2_ content increases, both the crystallization peak temperature and the activation energy for crystallization tend to rise, indicating that TiO_2_ acts as an effective nucleating agent that modifies the thermal stability and crystallization kinetics of the glass. This enhancement in nucleation promotes the formation of a higher volume fraction of uniformly distributed, fine-grained crystalline phases throughout the glass matrix. The presence of these homogeneously dispersed microcrystals not only refines the overall microstructure but also contributes to the improvement of the mechanical performance of the glass-ceramics, including their hardness, strength, and resistance to fracture. Consequently, the systematic addition of TiO₂ within the studied range (2.5–10 mol%) results in the development of structurally reinforced glass-ceramics with superior mechanical properties compared to TiO₂-free compositions^41^.

The formation of these crystalline phases, often with plate-like or interlocking microstructures, contributes to improved crack deflection and resistance, thereby increasing hardness and toughness^39–41^. The combined effect of network strengthening in the glass phase and enhanced crystallinity in the glass-ceramic phase explains the consistent increase in microhardness with TiO_2_ content. These results align well with previous studies^33–35,41,42^ showing that TiO_2_ not only acts as a network former but also as an effective nucleating agent that adjusts the crystallization behavior and microstructure, ultimately improving thermo-physical and mechanical properties. The data suggest that optimizing TiO_2_ content in lithium aluminosilicate glasses and glass-ceramics is a viable strategy to achieve materials with superior hardness and stability.

Such behavior aligns with multiple studies^12–14,43^ showing that these hardness values, especially in glass-ceramics, fall within ranges desirable for demanding applications such as dental restorations, where high surface hardness is essential to resist wear and maintain longevity. Indeed, microhardness is a key indicator of a material’s ability to withstand masticatory forces and abrasive oral environments, and TiO_2_-doped lithium aluminosilicate glass-ceramics demonstrate promising potential for dental use due to their enhanced stability and mechanical strength^12–14,43^. However, it is also noted from dental glass-ceramic studies that TiO_2_ improves mechanical properties effectively up to an optimal concentration (around 2.5 wt% in some systems), beyond which increased solubility and reduced flexural strength may occur, indicating the need for compositional optimization depending on the specific application.

The TiO_2_-doped glass and glass-ceramics prepared in this study show hardness values comparable to those of commercial lithium disilicate-based dental glass-ceramics, such as IPS e.max, which are favored in dentistry for their excellent combination of strength, esthetics, and wear resistance. The Vickers hardness of the studied samples is approximately 5.4 GPa, matching that of IPS e.max. This hardness is higher than that of feldspathic porcelain but lower than zirconia, which can reach about 12 GPa. The intermediate hardness of these materials provides an optimal balance, making them sufficiently strong to withstand wear and deformation during use, while not being overly hard, thereby reducing wear on opposing natural teeth.

### Compressive strength

**Fig. 8:**
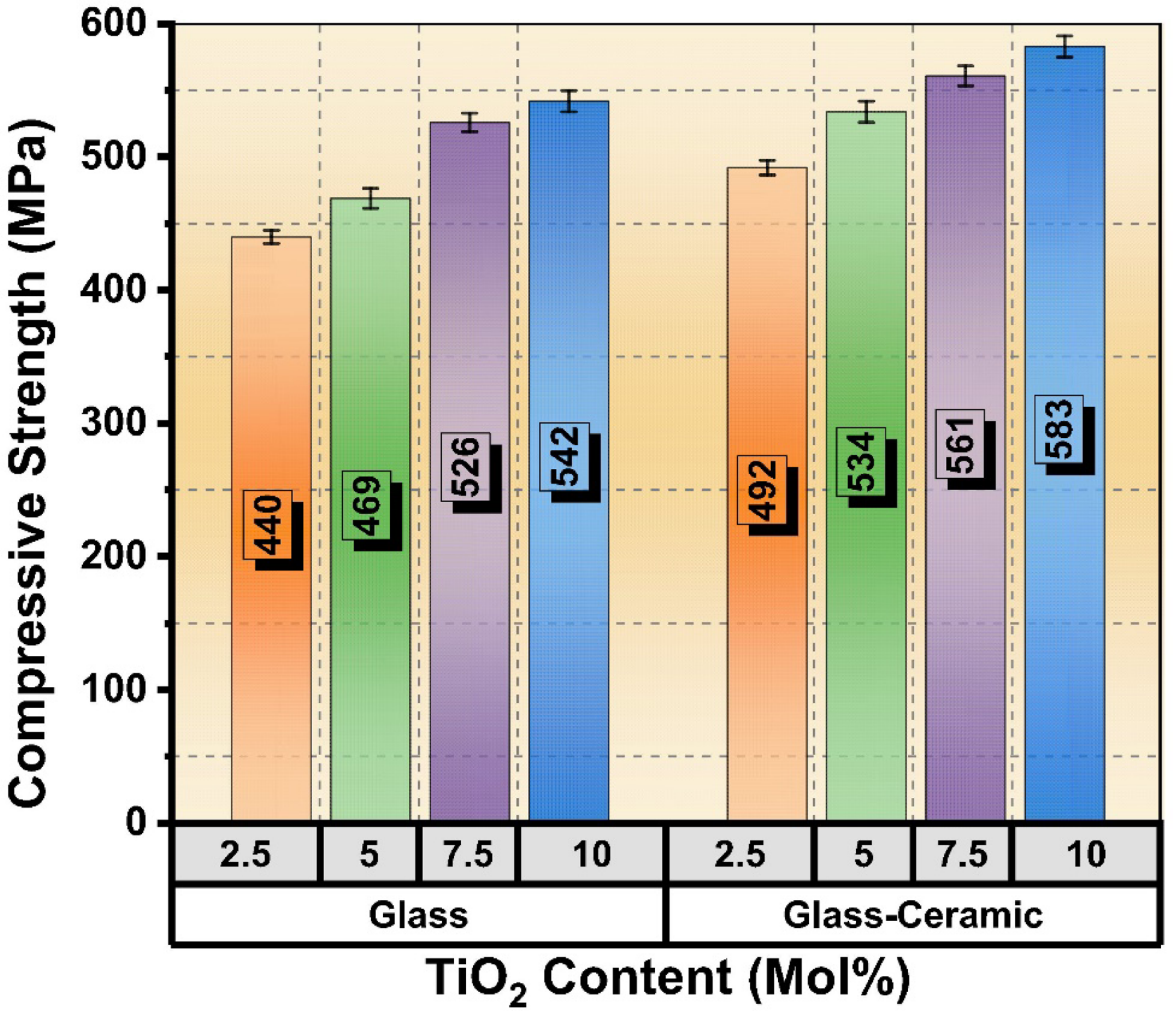
Compressive strength of the prepared glasses and their corresponding glass-ceramics.

Figure 8 depicts the compressive strength behavior and their values of contents 2.5, 5, 7.5, and 10 mol %, TiO_2_-doped lithium silicate glasses together with their corresponding glass-ceramics. The glass samples exhibited compressive strengths of 440, 469, 526, and 542 MPa, respectively. In comparison, the corresponding glass-ceramic samples showed higher compressive strengths of 492, 534, 561, and 583 MPa at the same TiO_2_ doping levels. The increase in compressive strength with higher TiO_2_ concentration can be attributed to the strengthening effect of titanium dioxide acting as a network modifier, which enhances the rigidity of the glass structure. Additionally, the glass-ceramic samples consistently displayed greater compressive strength than their glass counterparts due to the formation of crystalline phases during the controlled crystallization process^44,45^. These crystalline phases decrease porosity and increase material density, contributing to the mechanical enhancement. The improvement from glass to glass-ceramic is aligned with previous findings where TiO_2_ doping promotes nucleation and crystal growth, resulting in a more compact and stronger microstructure. The higher compressive strength of glass-ceramics indicates better resistance to deformation under load, which is critical for applications requiring mechanical stability.

Regarding the previous results, the synthesized TiO_2_-doped glass-ceramics hold significant promise for dental applications due to their enhanced mechanical strength, bioactivity, and biocompatibility. Their high compressive strength and hardness make them suitable for load-bearing uses such as dental implants, where stability and resistance to mechanical stress are critical. Additionally, the bioactive nature of these materials supports bone regeneration, making them excellent candidates for bone grafts and scaffolds in periodontal and maxillofacial reconstruction. The improved chemical stability and antimicrobial properties imparted by TiO_2_ doping also favor their use as dental fillers and restorative materials, including crowns and bridges, ensuring long-term performance and reduced risk of infection. Overall, TiO_2_-doped lithium silicate glass-ceramics can be effectively utilized in diverse dental applications where both mechanical integrity and biological compatibility are essential.

**Fig. 9:**
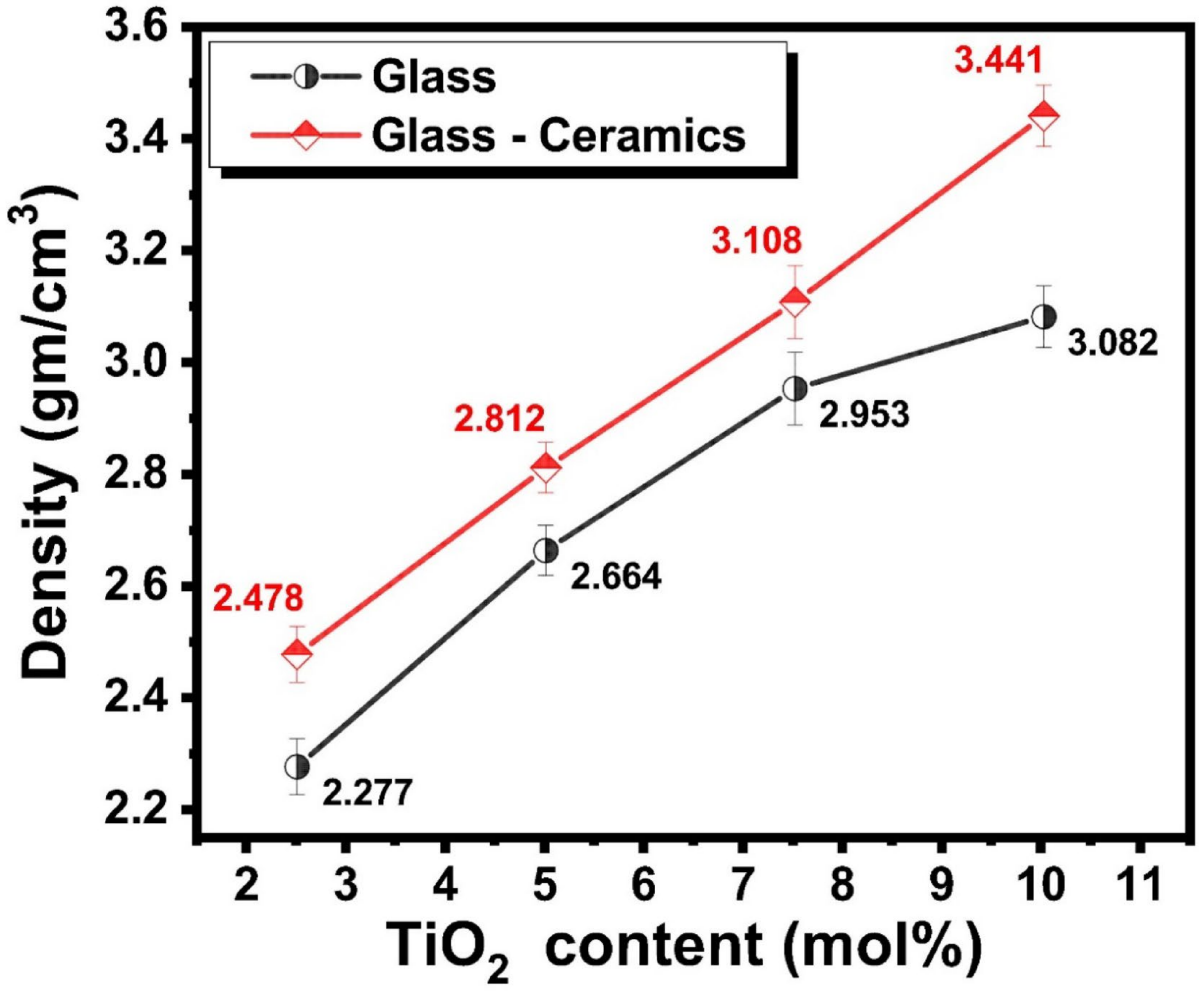
The density of the prepared glasses and their inverted glass–ceramics with different mol% of TiO_2_.

Figure 9 reveals the behavior of density values against the TiO_2_ content. The measured density values of the prepared glass samples were found to be 2.277, 2.664, 2.953, and 3.082 g/cm³ for samples containing 2.5, 5, 7.5, and 10 mol% TiO_2_, respectively. A similar trend was observed for the corresponding inverted glass-ceramics, which exhibited slightly higher densities of 2.478, 2.812, 3.108, and 3.441 g/cm^3^ at the same TiO_2_ concentrations. These results clearly demonstrate a progressive increase in density with increasing TiO_2_ content in both the glass and glass-ceramic systems. Regarding the previous study by Albarzan et al.^46^, the observed increase in density can be attributed to the incorporation of TiO_2_, a relatively heavy oxide (molecular weight ≈ 79.87 g/mol), into the glass network. As Ti^4+^ ions replace lighter network modifiers or occupy interstitial positions, the overall mass per unit volume of the glass increases. Furthermore, TiO_2_ acts as an intermediate oxide, capable of both modifying and forming the glass network. At lower concentrations, Ti^4+^ ions may enter the glass network by substituting for Si^4+^ ions, leading to a more compact structure. At higher concentrations, TiO_2_ promotes structural densification by forming Ti-O-Ti or Ti-O-Si linkages, which enhance the packing efficiency and reduce the free volume within the matrix. The slightly higher density values observed in the inverted glass-ceramics compared to their parent glasses are likely due to the partial crystallization that occurs during the heat-treatment process. The formation of crystalline phases such as Ti-containing compounds typically results in a more closely packed arrangement of ions, further reducing porosity and increasing the overall density. This trend indicates that crystallization enhances the structural compactness of the material.

### FTIR characteristics

The Fourier Transform Infrared (FTIR) analysis of the glass composition, formulated as 65SiO_2_ - (22.5-x) Li_2_O − 12.5Al_2_O_3_ - xTiO_2_ (where x = 2.5, 5, 7.5, and 10 mol%), demonstrates a notably complex vibrational behavior, as evidenced by the presence of broad absorption bands throughout the recorded spectrum. This broadness in the absorption features is a clear indication of the coexistence and overlapping of multiple vibrational modes that arise from the diverse structural units present within the glass network. These structural units encompass various bonding environments, including bridging and non-bridging oxygens, different coordination states of silicon, aluminum, and titanium, as well as the influence of modifier cations such as lithium. Because these vibrational modes overlap significantly, the resulting spectral bands are not sharply defined, making it challenging to assign specific bands to distinct molecular vibrations directly. Therefore, to accurately identify and characterize the individual vibrational contributions and gain a better understanding of the underlying glass structure, it is essential to apply spectral deconvolution techniques. These methods allow the separation of the composite broadband into its constituent peaks, enabling a more detailed and precise interpretation of the glass network’s vibrational dynamics and structural complexity.

**Fig. 10:**
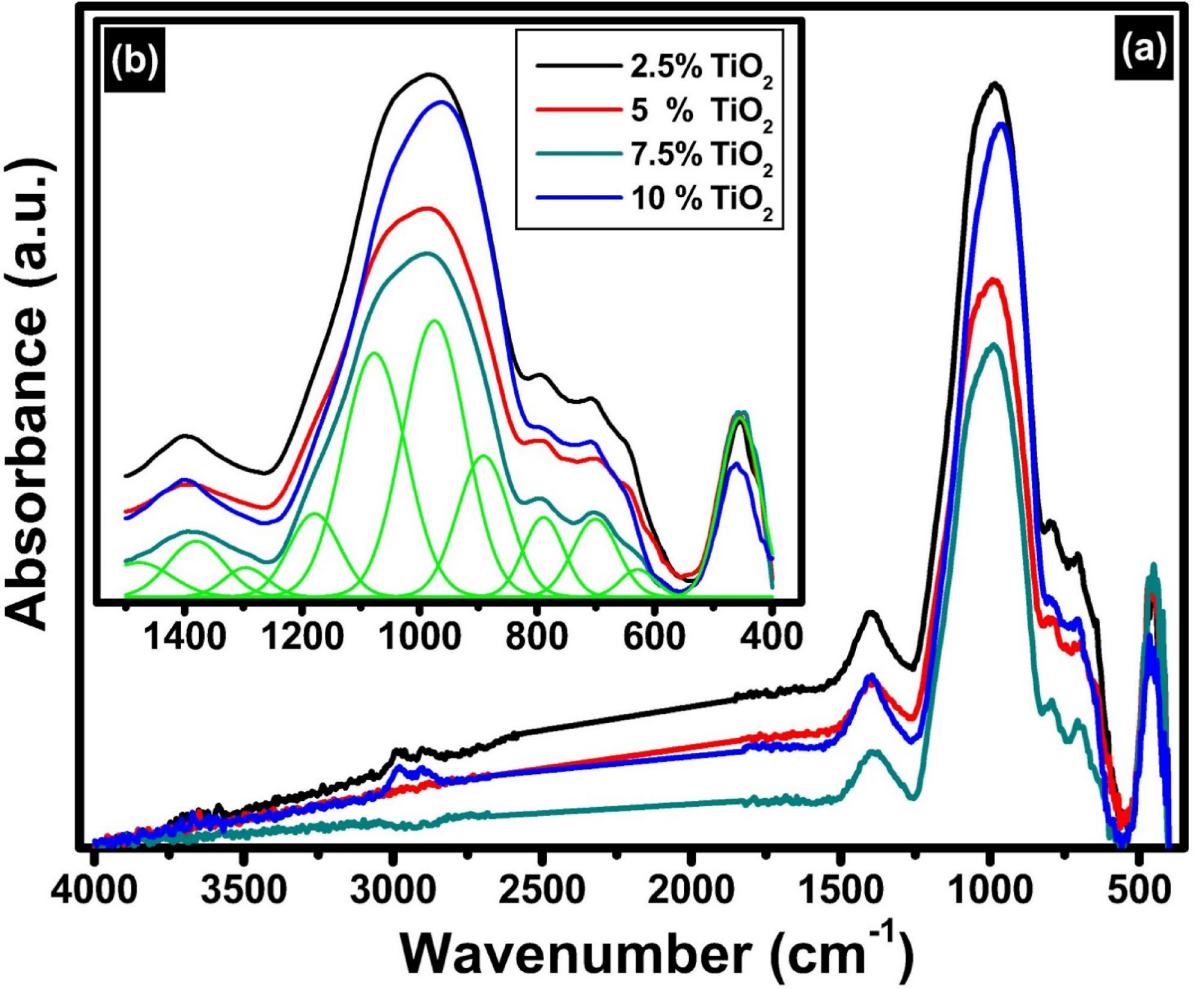
FTIR absorption spectra of the prepared TiO_2_-doped glasses, where (**a**) the full FTIR range and (**b**) deconvolution in the range 400–1400 cm^− 1^.

The FTIR spectrum presented in Fig. 10 reveals the presence of eleven clearly distinguishable absorption bands, each located at approximate wavenumbers of 450, 628, 698, 789, 892, 975, 1076, 1177, 1293, 1378, and 1469 cm^− 1^. These spectral features are indicative of specific vibrational modes that can be attributed to the structural components within the glass network. Specifically, these absorption bands are associated with the fundamental vibrations of silicate, aluminate, and titanate units, which form the primary framework of the glass structure. In addition to these network-forming units, the spectrum also reflects the influence of modifying cations, particularly lithium ions (Li^+^), which interact with the glass matrix and alter the vibrational environment. The presence and positions of these bands provide valuable insights into the molecular architecture and compositional complexity of the glass, highlighting the contributions of both the tetrahedral structural units and the modifying species to the overall spectral profile.

The assignments of the FTIR bands and their corresponding vibrational modes can be interpreted based on insights from previous research studies, as outlined below^47–55^.

(i) The low-frequency band at 450 cm^− 1^ is generally attributed to bending vibrations of Si-O-Si linkages or bending modes of Al-O bonds in the aluminosilicate framework. It may also include contributions from Ti-O vibrations in TiO_2_-containing glasses, reflecting the network connectivity and rigidity.
(ii) The bands at 628 and 698 cm^− 1^ are often assigned to symmetric bending or deformation modes of oxygen atoms bridging silicon and aluminum tetrahedra (Si-O-Al) or to vibrations involving Ti-O bonds in octahedral coordination. Additionally, these bands may include contributions from Ti-O vibrations, especially if titanium is incorporated into the glass network as TiO_6_ octahedra or TiO_4_ tetrahedra. The Ti-O bonds typically absorb in this region, and their presence can broaden and shift these bands, reflecting changes in the local environment and coordination of titanium.
(iii) The bands at 789 cm^-1^ and 892 cm^-1^ are generally associated with stretching vibrations of Si–O bonds in different structural environments. The 789 cm⁻¹ band can be linked to symmetric stretching vibrations of Si – O – Si linkages, whereas the 892 cm⁻¹ band often corresponds to vibrations involving non-bridging oxygens (NBOs) or Si – O – Li bonds formed due to the modifier effect of lithium oxide (Li_2_O). The presence and intensity of these bands provide insight into the degree of network depolymerization, where an increase in NBOs leads to more pronounced bands in this region.
(iv) The band centered at 975 cm^-1^ is a well-known signature of stretching vibrations of Si–O^-^ groups associated with non-bridging oxygens. This band is highly sensitive to the modifier content, as lithium ions break Si–O–Si bridges, creating NBOs that manifest as distinct vibrational modes. Changes in the intensity and position of this band with varying TiO_2_ content indicate alterations in the glass network connectivity and the balance between bridging and non-bridging oxygens.
(v) The bands at 1076 cm^-1^ and 1177 cm^-1^ are characteristic of asymmetric stretching vibrations of bridging oxygens in Si - O - Si and Si - O - Al linkages. The presence of aluminum substituting for silicon in the tetrahedral network introduces Si - O - Al bonds, which shift and broaden these bands due to differences in bond strength and mass. The 1177 cm⁻¹ band, in particular, is often attributed to Si–O–Al asymmetric stretching, indicating the extent of aluminum incorporation and its impact on network polymerization.
(vi) The band at 1293 cm^-1^ is less commonly observed but can be associated with overtone or combination bands involving Si-O stretching modes or vibrations related to Ti-O bonds in tetrahedral coordination. Titanium incorporation into the glass network can induce shifts and splitting in this region due to changes in local symmetry and bonding environments.
(vii) The bands at 1378 cm^-1^ and 1469 cm^-1^ are generally assigned to bending vibrations involving modifier cations such as lithium and their interactions with oxygen atoms. In some cases, these bands may also correspond to Li – O bending modes or complex vibrational modes involving aluminum in higher coordination states.

**Fig. 11:**
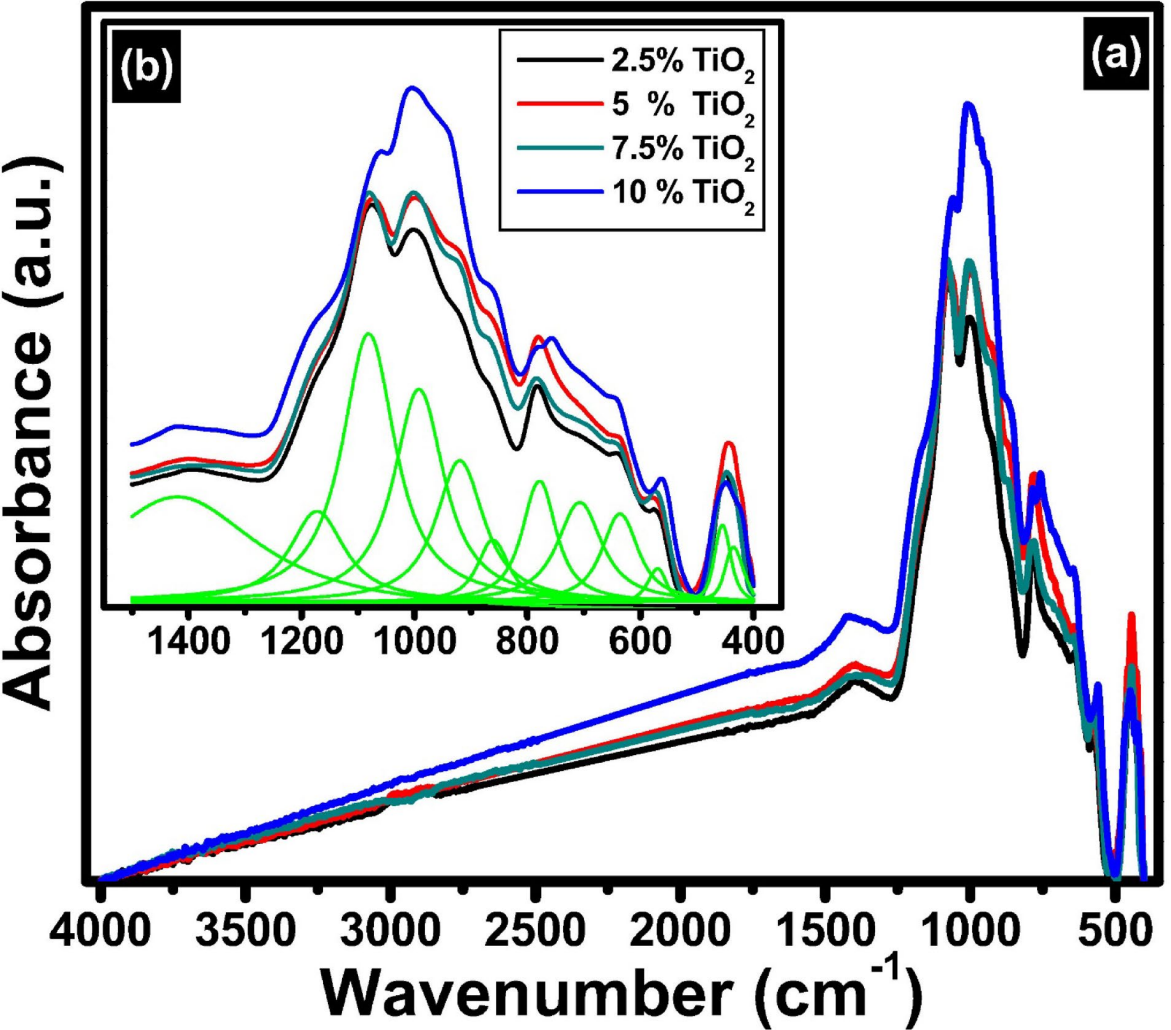
FTIR absorption spectra of the prepared TiO_2_ doped glass – ceramics, where (**a**) the full FTIR range and (**b**) deconvolution in the range 400–1400 cm^− 1^.

On the other hand, the FTIR spectrum of the glass-ceramics derived is represented in Fig. 11 reveals a more complex and well-resolved vibrational profile compared to the parent glass, characterized by 12 distinct absorption bands centered at approximately 435, 457, 564, 634, 703, 776, 861, 921, 990, 1078, 1176, and 1413 cm^− 1^. This increased number of bands and their sharper features reflect the structural reorganization and crystallization processes occurring during the glass-to-glass-ceramic transformation, which leads to the formation of ordered crystalline phases and a reduction in structural disorder. The identification of FTIR bands of the corresponding glass-ceramics and their related vibrational modes can be understood by considering information from prior investigations, as shown in the following references^47–55^.

(i) The bands observed at 435 and 457 cm^-1^ correspond to bending vibrations of Si – O – Si and Al – O – Si linkages as well as lattice vibrations associated with emerging crystalline silicate or aluminosilicate phases, indicating multiple distinct local environments due to phase separation or crystallite formation.
(ii) The band at 564 cm^-1^ is particularly significant as it is assigned to Ti – O stretching vibrations in TiO_6_ octahedra, confirming that titanium ions adopt more ordered coordination geometries within the crystalline lattice, contrasting with their more disordered state in the glass. The 634 cm^-1^ band is attributed to symmetric bending modes of bridging oxygens in Si – O – Si or Si – O – Al linkages, with its sharper nature signifying increased network connectivity and crystallinity.
(iii) Mid-frequency bands at 703 and 776 cm^-1^ are linked to Si – O stretching vibrations in crystalline silicate structures, often associated with Q^3^ species (silicon tetrahedra with three bridging oxygens), which indicate a higher degree of polymerization in the glass-ceramic relative to the parent glass. The bands at 861, 921, and 990 cm⁻¹ correspond to non-bridging oxygen vibrations or Si – O – Li bonds and asymmetric stretching of Si – O – Si and Si – O – Al linkages, respectively, and their increased intensity and sharpness reflect the formation of well-defined silicate frameworks and lithium-containing crystalline phases such as lithium aluminosilicates.
(iv) Higher frequency bands at 1078 and 1176 cm^-1^ are characteristic of asymmetric stretching vibrations of bridging oxygens in Si – O – Si and Si – O – Al bonds, further evidencing the enhanced network polymerization and aluminum substitution within the crystalline matrix.
(v) The band at 1413 cm^-1^ is attributed to bending vibrations involving modifier cations like lithium, indicating complex vibrational modes arising from lithium incorporation into crystalline phases or remaining amorphous regions.

### The impacts of TiO_2_ and crystallization

The addition of TiO_2_ and subsequent heat treatment significantly influence the structural and physical characteristics of the prepared glass and its corresponding glass-ceramics. In the FTIR spectra, the broadening of bands before deconvolution reflects the structural complexity introduced by various network formers and modifiers, including SiO_4_, AlO_4_, TiO_4_/TiO_6_ units, and Li^+^ ions. As TiO_2_ is incorporated and increasing content its content from 2.5 mol% to 10 mol%, Ti – O vibrational modes emerge in the mid-IR range, particularly overlapping with Si – O and Al – O stretches in the 600–700 cm^− 1^ and 1200–1300 cm^− 1^ regions, leading to noticeable band broadening and shifts. These spectral changes indicate alterations in network connectivity, as Ti can either enhance polymerization when acting as a network modifier. Concurrently, the reduction in Li_2_O content, which functions as a network modifier, decreases the number of non-bridging oxygens (NBOs), especially evident in the diminishing intensity of the band near 975 cm^− 1^. This shift reflects an increase in network polymerization, which plays a crucial role in the thermal stability and crystallization behavior during heat treatment. The presence of Al_2_O_3_ further affects the network structure by introducing tetrahedrally coordinated aluminum, which substitutes for Si and requires charge compensation from Li⁺, altering the Si – O – Al linkages and shifting bands around 1076 and 1177 cm^− 1^. Structural changes in TiO_2_ detected by FTIR show variations in Ti-O-Ti bonding, which influence crystallinity observed by XRD after heat treatment. Increasing TiO_2_ content promotes nucleation and growth of crystalline phases, resulting in sharper XRD peaks typical of anatase or rutile structures. FTIR identifies shifts in bonding environments, while XRD confirms crystalline phase development and increased order. Together, they reveal how thermal processing transforms the glass from amorphous to crystalline, with higher TiO_2_ favoring crystal growth. The FTIR band positions and their assignments are concluded in Table 1.

Regarding the previous interpretations, TiO₂ acts as an effective nucleating agent in glass systems by lowering the energy barrier for crystallization and altering the silicate glass network. It disrupts Si-O-Si bonds to form Si-O-Ti linkages, leading to weaker structures that favor nucleation. Ti ions transition from four-fold to six-fold coordination within TiO_6_ units, serving as structural templates for crystal formation. Titanium-rich regions created by TiO_2_ enhance nucleation, reduce activation energy, and promote desirable crystalline phases during heat treatment. Overall, TiO_2_ facilitates controlled crystallization by modifying network structure, promoting surface nucleation, and stabilizing preferred crystalline phases in glass-ceramics.

The evolving microstructure, as revealed by SEM, transitions from an amorphous matrix to well-defined crystalline domains, corresponding to enhanced hardness values due to the densification and reinforcement of the glass network. Thus, the compositional changes driven by TiO_2_ addition and thermal processing govern the structural evolution, phase development, and mechanical performance of both the glass and its glass-ceramic derivatives.

**Table 1:** Identified vibrational peaks in the studied glasses and their assignments^40–48^.

Band position wavenumber (cm^− 1^)	Assignments
450–564	Bending vibrations of silicate Al – O Ti - O
628–698	Si – O – Al Ti – O (TiO_4_/TiO_6_)
703–789	Si – O – Si
892	NBO (Si – O – Li)
975–990	Si – O^−^
1076–1177	Asymmetric stretching vibrations Si – O – Si
1292	Stretching vibrations Si – O Ti – O
1378–1469	Li – O bending vibrations

## Conclusions

A series of glass samples with the nominal composition 65SiO_2_ - (22.5 - x)Li_2_O − 12.5Al_2_O_3_ – xTiO_2_ (where x = 2.5, 5, 7.5, and 10 mol%) were synthesized via a conventional melt-quenching technique, and their glass–ceramic derivatives were developed based on DSC data. Differential scanning calorimetry revealed an endothermic peak around 495 °C followed by an exothermic peak near 700 °C, indicating a clear transition from amorphous to crystalline phases within a stable thermal window suitable for processing; notably, doping with 10 mol% TiO_2_ shifted these peaks to higher temperatures (502 °C and 720 °C), reflecting altered thermal behavior. X-ray diffraction analysis identified three primary crystalline phases, lithium disilicate (Li_2_Si_2_O_5_), lithium aluminosilicate (LiAlSiO_4_), and brookite (TiO_2_), with lithium disilicate exhibiting strong, sharp peaks indicative of high crystallinity, while brookite appeared predominantly at higher TiO_2_ concentrations, suggesting the role of TiO_2_ in phase evolution. Microstructural examination revealed a dense network of interlocking lath-like crystals that evolve into square pyramid-like shapes at higher magnifications, demonstrating a controlled, directional crystallization process; increasing TiO_2_ content promoted the growth of larger, sharper crystals with well-defined boundaries, confirming its function as an effective nucleating agent that refines morphology and enhances structural integrity. EDAX confirmed the presence of oxygen, silicon, aluminum, and titanium, with incorporation of titanium substantiating its influence on crystal growth and crystallization enhanced with TiO_2_ content. Microhardness measurements showed a progressive increase from 5.02 to 5.93 GPa with rising TiO_2_ content in the glasses, the progressive increase in TiO_2_ from 2.5 to 5% yields a approximately 7% increase in the hardness this can be attributed to TiO_2_ acting as a network former or intermediate oxide that increases cross-link density and reduces non-bridging oxygens, thereby strengthening the glass network; subsequent heat treatment further enhanced microhardness to between 5.51 and 7.27 GPa due to the nucleating effect of TiO_2_ facilitating the formation of hard crystalline phases such as lithium disilicate and lithium aluminosilicate. FTIR Spectroscopic analysis revealed that the addition of network modifiers like Li_2_O and TiO_2_ disrupts the SiO_4_ tetrahedral framework by breaking Si – O – Si bridging bonds and generating non-bridging oxygens (NBO), leading to network depolymerization and reduced rigidity, which is reflected in shifts in vibrational spectra and impacts the mechanical properties of the material. Taken together, these findings demonstrate that the tailored incorporation of TiO_2_ not only controls crystallization and microstructure but also significantly enhances mechanical strength and stability, showing potential for dental applications due to their optimized combination of structural integrity, hardness, and thermal stability.

## Data Availability

The datasets generated and/or analyzed during the current study are not publicly available because they are private, but are available from the corresponding author on reasonable request.
